# BETTER DIET QUALITY IS ASSOCIATED WITH REMAINING NON-FRAIL OVER TIME IN A DIVERSE COHORT

**DOI:** 10.1093/geroni/igad104.3202

**Published:** 2023-12-21

**Authors:** Marie Kuczmarski, May Beydoun, Michael Georgescu, Nicole Noren Hooten, Nicolle Mode, Michele Evans, Alan B Zonderman

**Affiliations:** University of Delaware, Newark, Maryland, United States; National Institute on Aging, Baltimore, Maryland, United States; National Institute on Aging, Baltimore, Maryland, United States; National Institute on Aging, Baltimore, Maryland, United States; National Institute on Aging, Baltimore, Maryland, United States; National Institute on Aging, Baltimore, Maryland, United States; National Institute on Aging, Baltimore, Maryland, United States

## Abstract

Frailty is a major public health concern affecting the health and well-being of adults. Poor diet is among several modifiable risk factors for developing frailty. We examined associations of dietary quality assessed by the Healthy Eating Index with frailty status over mean 8.7y follow-up. The sample was comprised of African American (AA) and White participants enrolled in the Healthy Aging in Neighborhoods of Diversity across the Life Span (HANDLS) study (44% male, 44% AA, mean baseline age of 48.5y). Only participants from the baseline study and first 2 follow-up visits with dietary data, 2 recall days per visit, and frailty data were included (n=2901). Frailty was prospectively determined by the modified FRAIL scale. Group-based trajectory modeling identified 3 diet quality groups (high: g3 [5.2% of sample, HEI(X±SD): 57.4±4.1], medium (g2: 48.2%, 46.1±3.7), low (g1: 46.5%, 38.2±2.9) and 2 frailty groups (remaining non-frail [20.2% sample] or being pre-frail or frail over time). Multiple logistic and Cox proportional hazards regressions found high and medium HEI trajectory groups, compared to the low HEI group, were associated with reduced risk of being pre-frail or frail, with a clear dose-response relationship (g2 vs. g1: HR=0.77, 95% CI: 0.64-0.92, p=0.004; g3 vs. g1: HR=0.29, 95% CI: 0.18-0.49, P< 0.001), even after adjusting for sociodemographic factors, smoking, drug use, and allostatic load. These findings were corroborated with Kaplan-Meier curves and log-rank tests, providing longitudinal evidence of the link between better diet quality with lower frailty risk, and the importance of diet in successful aging during middle age.

